# Effect of *Lactobacillus rhamnosus* on Physicochemical Properties of Fermented Plant-Based Raw Materials †

**DOI:** 10.3390/foods10030573

**Published:** 2021-03-10

**Authors:** Carmen Masiá, Asger Geppel, Poul Erik Jensen, Patrizia Buldo

**Affiliations:** 1 Department of Food Science, University of Copenhagen, Rolighedsvej 26, 1958 Frederiksberg, Denmark; cala@food.ku.dk (C.M.); peje@food.ku.dk (P.E.J.); 2 Chr. Hansen A/S, Bøge Alle 10-12, 2970 Hørsholm, Denmark; dkage@chr-hansen.com

**Keywords:** fermentation, plant-based, LAB, *L. rhamnosus*, rheology, flavor, sensory

## Abstract

To overcome texture and flavor challenges in fermented plant-based product development, the potential of microorganisms is generating great interest in the food industry. This study examines the effect of *Lactobacillus rhamnosus* on physicochemical properties of fermented soy, oat, and coconut. *L. rhamnosus* was combined with different lactic acid bacteria strains and *Bifidobacterium*. Acidification, titratable acidity, and viability of *L. rhamnosus* and *Bifidobacterium* were evaluated. Oscillation and flow tests were performed to characterize rheological properties of fermented samples. Targeted and untargeted volatile organic compounds in fermented samples were assessed, and sensory evaluation with a trained panel was conducted. *L. rhamnosus* reduced fermentation time in soy, oat, and coconut. *L. rhamnosus* and *Bifidobacterium* grew in all fermented raw materials above 10^7^ CFU/g. No significant effect on rheological behavior was observed when *L. rhamnosus* was present in fermented samples. Acetoin levels increased and acetaldehyde content decreased in the presence of *L. rhamnosus* in all three bases. Diacetyl levels increased in fermented oat and coconut samples when *L. rhamnosus* was combined with a starter culture containing *Streptococcus thermophilus* and with another starter culture containing *S. thermophilus*, *L. bulgaricus* and *Bifidobacterium*. In all fermented oat samples, *L. rhamnosus* significantly enhanced fermented flavor notes, such as sourness, lemon, and fruity taste, which in turn led to reduced perception of base-related attributes. In fermented coconut samples, gel firmness perception was significantly improved with *L. rhamnosus*. The findings suggest that *L. rhamnosus* can improve fermentation time and sensory perception of fermented plant-based products.

## 1. Introduction

Favorable organoleptic properties dominate over sustainability or health in consumer behavior [1]. Texture and flavor attributes play an important role in plant-based products [2] and their improvement is essential for successful product development. This can be achieved through fermentation [3], enabling the development of clean label products and avoiding excessive food processing, or the use of additives [4]. In this context, it is crucial to understand the effect that starter cultures have on plant matrices. Soybean products have dominated for the past few decades [5,6], but the portfolio of plant-based raw materials has expanded into other legumes, nuts, seeds, pseudocereals, and cereals [7]. Soy [7,8,9], oat [10], and coconut [11] are some of the most popular substrates due to their nutritional value and/or their physicochemical properties.

Soy milk has a high protein content (2.5–3.1 g per 100 mL [12]) and high levels of non-digestible oligosaccharides (NDOs). Fermentation with lactobacilli has been proven to metabolize soy NDOs [13], and also to contribute to the rheological characteristics of soy-based gels [14,15]. Soy protein gelation is the main feature in fermented soy gels [16] and it is affected by different factors, such as globulin ratios [17] in protein–polysaccharide blends and the molecular weight of polysaccharides [16]. Moreover, starter cultures could influence textural properties of soy gels. For instance, higher viscosity was observed with binary culture fermentation, specifically with a combination of *L. acidophilus* and *L. plantarum* [18]. Fermented soy is associated with a characteristic beany flavor caused by lipid oxidation products (mostly pentanal and n-hexanal [19]). However, this can be overcome by deactivating the enzymes that produce off-flavors through different methods, such as pulse electrification, high-pressure processing, or ohmic heating [5].

Oats have gained popularity lately due to their dietary fibers, namely β-glucan soluble fibers found in endosperm cell walls. They are considered prebiotics and serve as feed for intestinal microflora [20]. Oat groats contain 15–20% protein with a complete amino acid profile [21] and its digestibility is generally high [22]. However, commercial oat-based milks present a protein deficiency (≤1 g per 100 mL of product) [8]. This questions their suitability as a substrate for yogurt- and cheese-like products, since proteins are an essential factor for texture formation in fermented materials [16,23]. Nevertheless, this can be solved with protein supplementation or by preparing a matrix from an oat concentrate. Globulins are the main proteins in oat and they have a similar structure to soy 11S protein, also aggregating under 100 °C [24]. The major component of oat carbohydrates is starch (40–60%) [25], which enhances rheological properties of fermented products, contributing to gelation. Oat lipid oxidation is responsible for the off-flavor of oat-based products [26]. Volatile compounds, such as hexanal, pentanal, and certain carbonyl compounds [27,28] give oat drinks an unpleasant aftertaste related to rancidity. Additionally, the presence of long-chain hydroxy fatty acids causes bitter off-notes in oat flavors [29]. These negative attributes should be masked or removed to achieve consumer acceptance of an oat-based dairy alternative.

Coconut is another popular raw material for the development of plant-based products. Commercial coconut milk has ≤1 g of protein and 4–6 g of fat per 100 g of product [12,30]. Its high saturated fatty acids have a small melting temperature range (18.3–26.4 °C [31]) influenced by short-chain lauric acid [32]. Its ability to form solid structures at an ambient temperature makes it a good candidate to obtain firm products, such as non-dairy yogurt or non-dairy cheese, but due its lack of protein and high saturated fat content, it is not the most optimal replacement for dairy alternatives [12]. Coconut milk contains a low carbohydrate content (5.5 g/100 g of product) that can be reduced to 1.32 g/100 g of product after fermentation [33]. Previous studies highlight coconut milk as a favorable medium for probiotic bacterial growth [11] and for cell viability preservation after fermentation [6].

The potential of lactic acid bacteria (LAB) is of great interest for plant-based fermentations. They can produce an extended portfolio of aromatic compounds, endowing fermented products with characteristic flavors and aromas originating with the breakdown of the predominant macronutrients in the food matrix [34]. Certain LAB can produce exopolysaccharides (EPS), which improve texture in fermented products, increasing viscosity and improving stability [35]. This feature can enable the replacement of thickening agents. *L. delbrueckii* [36], *S. thermophilus*, *L. delbrueckii* subsp. *bulgaricus* [9], *B. longum* or *L. rhamnosus* [37] have already been used in the development of fermented plant-based dairy alternatives. Plant-based fermentation with pure and mixed cultures has been evaluated [38]. It was found that both are suitable for oat fermentation, but pure cultures showed better flavor profiles. In the current study, different cultures were combined with *L. rhamnosus*, LGG^®^, a registered trademark of Chr. Hansen A/S identifying *L. rhamnosus* strain and related products. Previous studies showed that *L. rhamnosus* can grow in bases containing cereals and pseudocereals [10,39,40]. It is able to produce exopolysaccharides (EPS) in dairy milk [41], which characterizes it as a functional starter culture [42]. Its functionality has been previously studied in plant-based raw materials [43], and it was shown that EPS production takes place at lower temperatures than growth temperature in oat-based media, which indicates that EPS are produced after fermentation [44]. Their contribution to product viscosity [45,46] and prevention of phase separation in oat yogurts fermented with EPS-producing strains was also highlighted. Polysaccharide production is conditioned by the presence of different sugars in varying amounts in initial raw materials [47]. Lactose, galactose, and glucose were remarked as the most efficient carbon sources for EPS production with *L. rhamnosus* [47]. This strain has also been used in studies that involve the fermentation of different legumes [39], cereals [40], coconut, and hemp [6]. All studies reported its ability to grow and acidify, and no negative effects on its viability or on the sensory properties of the final products. Nevertheless, further research needs to be carried out for a deeper understanding of probiotics in plant-based fermentation. Another probiotic with great potential for plant-based fermentation is BB-12^®^ *Bifidobacterium*, also a trademark of Chr. Hansen A/S. Satisfactory growth of BB-12^®^ in fermented soy with different carbohydrate content has been reported [48]. In addition, α-galactosidase activity of BB-12^®^ can reduce galacto-oligosaccharide levels in soy milk. Viability of BB-12^®^ was likewise shown in a soy dessert during 6 months of storage at colony-forming units (CFU) levels above 10^7^ CFU/g [1]. It has been suggested that soy milk and coconut milk could be richer media for BB-12^®^ growth in comparison to dairy milk due to their amino acids profile [49].

The aim of this study was to understand the effect of LGG^®^ in combination with different bacterial cultures, including BB-12^®^, on physicochemical properties of different plant bases. Soy, oat, and coconut were selected as substrates to cover a broad spectrum of plant-based raw materials: legumes, cereals, and high-fat substrates, respectively. In addition, the synergy of LGG^®^ and different starter cultures, including BB-12^®^, and their acidification capacity and growth was assessed. It was intended to test if both probiotic strains could grow in plant materials above 10^7^ CFU/g, overtaking the recommended levels for a beneficial effect on human intestinal health (10^6^ CFU/g) [50,51].

## 2. Materials and Methods

For each base, three separate batches were produced; for each batch, duplicate measurements were taken in all the experiments.

### 2.1. Preparation and Fermentation of Plant Bases

Raw materials used for the preparation of the bases were soy milk (Naturli Foods, Vejen, Denmark), coconut milk (Aroy-D, Thai Agri Foods, Samut Prakan, Thailand), oat concentrate (Oatvita, Frulact, Tortosendo, Portugal), sucrose (Nordic Sugar, København, Denmark), and starch (Clearam CJ5025, Roquette, Frankfurt am Main, Germany). Media preparation was performed following the composition shown in Table 1. Bases were previously optimized to represent commercial plant-based products. Ingredients were mixed until homogeneous matrices were obtained.

Sample size for fermentation oscillated between 3 and 5 liters. Soy, oat, and coconut bases were pasteurized at 90 °C for 20 min and cooled to fermentation temperature (43 °C). The cultures used for the fermentation of the plant bases were provided by Chr. Hansen A/S (Hørsholm, Denmark) and they are shown in Table 2. Starter cultures were frozen DVS, and LGG^®^ was freeze-dried DVS. Samples were inoculated according to Chr. Hansen’s recommendation, namely, 0.02% of each starter culture separately, and also a combination of each starter culture with LGG^®^. A total of six different culture combinations were inoculated in each base. pH was measured with iCinac (AMS S.R.L., KPM Analytics, Rome, Italy) until samples reached pH 4.5. Afterwards, coagulum was broken with a perforate disc and a cooling and a smoothing process (25 °C and two bars back-pressure) were applied. Samples were stored at 6 °C.

#### 2.1.1. Post Acidification and Titratable Acidity during Storage

Post acidification of fermented samples was measured with a pH-meter (Mettler Toledo, OH, USA) at day 1, 7, 14, and 21. Acidimetric titration of fermented samples was performed by auto-titrator InMotion Pro (Mettler Toledo, OH, USA). A total of 5–15 g of fermented product was mixed with demineralized water in a disposable titration breaker (Mettler Toledo, OH, USA) up to 60 g without stirring. Samples were titrated against a 0.1 M sodium hydroxide (NaOH) solution and analyzed in duplicates. The equation to calculate titratable acidity (TA) values was as follows:

(1) $$TA\left(\%\;lactic\;acid\right)\;=\;\frac{\left(mL\;0.1NNaOH\times 0.9\right)}{g\;sample}$$

The molecular weight of lactic acid (90.08) is used as a constant in the equation (0.9). Buffer solutions (pH 4.01, pH 7, and pH 9.21 at 20 °C) used for titratable acidity and post-acidification pH measurements were provided by Hamilton Nordic AB (Kista, Denmark) and NaOH was obtained from VWR (Søborg, Denmark).

#### 2.1.2. Bacterial Survival during Storage

The CFU count of LGG^®^ was performed on De Man, Rogosa, and Sharpe (MRS) 6.5 agar media (BD Difco, NJ, USA) with 500 ppm of vancomycin (internal solution). The first dilution was prepared with 5 g of each sample in 45 g of sterile peptone saline diluent (Oxoid, Roskilde, Denmark) in sterile stomacher bags. Samples were mixed during 1 min at 230 rpm to ensure homogeneity, and serial 10-fold dilutions were prepared. 1 mL of each dilution was inoculated in empty Petri dishes, and 12–15 mL of previously prepared melted MRS agar media with vancomycin for LGG^®^ count was poured into the Petri dish and merged with the inoculum. The CFU count of BB-12^®^ was performed on MRS 6.5 agar media with 1 mL of cystein hydrochloride 10% (CyHCl) (Merck, Søborg, Denmark), and 1500 ppm of lithium mupirocin (MUP) (Sigma Aldrich,Søborg, Denmark). The same procedure as for LGG^®^ was performed for the BB-12^®^ count. Diluted samples and growth media were mixed, and all Petri dishes were incubated at 37 °C for 3 days under anaerobic conditions. Plates with 15 to 300 colonies were selected for cell count. Results were reported as CFU per gram.

### 2.2. Physicochemical Analysis of Fermented Bases

#### 2.2.1. Rheological Measurements

Rheological measurements were performed after 7 days of storage. Oscillation and flow tests were performed with a rheometer MCR 302 (Anton Paar GmbH, Graz, Austria). A stainless-steel coaxial cylinder (CC27 system, stator inner radius 28.9 mm, rotor outer radius 26.7 mm, height 40 mm, gap 1.130 mm) was used. An oscillation test was performed between 0.5–8 Hz at constant strain, and the complex modulus (G*), storage modulus (G${}^{\prime }$), and loss modulus (G${}^{\prime \prime }$) were measured. Complex modulus results at a frequency of 1.52 Hz were used for the statistical comparison of all samples based on a previous internal study. A flow test was performed with shear rates (γ) from 10^−3^ s^−1^ to 300 s^−1^ for the up-flow and from 300 s^−1^ to 10^−3^ s^−1^ for the down-flow. Shear stress (τ) was measured for each sample. Shear stress values for 45.2 s^−1^ shear rates were statistically compared, according to previous findings about oral perception of thickness being strongly correlated to deformation measurements for viscosity at a shear rate of 50 s^−1^ [52]. The hysteresis loop area between the up-flow and down-flow curves was also calculated.

### 2.3. Identification of Volatile Organic Compounds (VOCs)

#### 2.3.1. Identification of Targeted VOCs

Chemicals used for dilution and acidification were 1-methyl-2-pyrrolidone, Milli-Q Water (MQW), and 2M sulfuric acid. A 500/1000/5000 parts per million (ppm) stock solution of acetone (100 mg), acetaldehyde (100 mg), ethyl acetate (100 mg), 3-methyl-butanal (50 mg), ethanol (100 mg), diacetyl (100 mg), butan-1-ol (50 mg), and acetoin (1000 mg) were weighed accurately into a 100 mL measuring flask, filled to the mark with 1-methyl-2-pyrrolidone and mixed thoroughly. A 5/10/50 ppm standard solution was made by pipetting 5 mL of the stock solution to 495 mL MQW in a measuring flask and mixed carefully. The acetoin standard solution was analyzed separately, as it contains small amounts of diacetyl. Standard solutions and samples were prepared by adding 1 mL into a 20 mL headspace vial already containing 200 μL 2M sulfuric acid and sealed with teflon-lined aluminum caps, and analyzed on the day of preparation. The analysis of targeted VOCs was performed with a static headspace sampler connected to a Gas Chromatograph with Flame Ionization Detector (GC-FID) (Perkin Elmer, MA, USA), equipped with an HP-FFAP column, 25 m × 0.2 mm × 0.33 μm (Agilent Technologies, Glostrup, Denmark), using helium as carrier gas. Before injection of an aliquot of the headspace above the sample, the vial was incubated for 37 min at 70 °C. The GC-oven program was as follows: 60 °C/2 min, Ramp 1: 45 °C /min to 230 °C; Hold 0.5 min. Identification of VOCs was based on retention time in comparison with that of the standards. Calculation of the concentration of each compound was based on the peak height divided by the response factor (Equation (2)). The response factor was previously established with standard solutions by the quotient of the peak height divided by the known sample concentration.

(2) $$Sample\;concentration\;=\;\frac{Peak\;height}{Response\;factor}$$

#### 2.3.2. Identification of Untargeted VOCs

Dynamic Headspace Extraction Gas Chromatography and Mass Spectrometry (DHE-GC-MS) was performed using the same sample preparation as for targeted VOCs. A Multi-Purpose Sampler (Gerstel Gmbh, Mühlheim an der Ruhr, Germany), was performing DHE using a Tenax-TA Thermal Desorption Unit (TDU) tube (Gerstel#020810-005-00). Vials were incubated at 30 °C and VOCs extracted onto the Tenax TA TDU tube using 400 mL helium at 40 mL/min and subsequently dried using 600 mL helium at 60 mL/min. The Tenax TA tube was then inserted into the injection port consisting of a TDU on top of a CIS4 having a Tenax TA liner (Gerstel#012438-010-00) installed, which was kept at 10 °C during desorption of the Tenax TA TDU tube at 270 °C. Thereafter, the CIS liner was heated rapidly to 270 °C and VOCs transferred to the GC-column in splitless mode. The GC was equipped with a DB-5MS UI column, 30 m × 0.25 mm × 1 µm (Agilent Technologies, Glostrup, Denmark). The GC-oven program was as follows: 32 °C/2 min; Ramp 1: 10 °C/min to 102 °C, Hold 0 min; Ramp 2: 5 °C/min to 145 °C, Hold 0 min; Ramp 3: 15 °C/min to 200 °C, Hold 0 min; Ramp 4: 20 °C/min to 325 °C, Hold 0 min; Total run time: 27.5 min. The column was coupled to a 5977A MSD, Agilent Technologies, MS-detector in scan mode (m/z 29-209/3.9 Hz, ion source temperature = 230 °C). VOCs were tentatively identified using Retention Index and NIST library search (NIST MS search program 2017 release, National Institute of Standards and Technology, Gaithersburg, MD, USA). Feature extraction as heights, retention time, and noise was done using MassHunter Quantitative Analysis software v.10.1 (Agilent Technologies, Glostrup, Denmark), and final results were reported as signal-to-noise (Equation (3)).

(3) $$S/N\;=\;\frac{Height_{analyte}}{Noise_{analyte}}$$

Chemicals for targeted and untargeted VOCs identification were obtained from Sigma–Aldrich (Munich, Germany), except from 1-Methyl-2-pyrrolidone, which was purchased from Merck (Merck KGaA, Darmstadts, Germany).

### 2.4. Sensory Analysis

Sensory evaluation was performed according to the International Organization for Standardisation [53]. The sensory lab was equipped with standardized individual booths, white light, and controlled temperature. Descriptive analysis of fermented samples was performed by a trained sensory panel of nine trained panelists. A training session took place before the tasting session to familiarize the panelists with the samples and to define the attributes to be evaluated. Three different sessions took place, one for each fermented base, and six samples were presented per session, corresponding to the six different culture combinations. Samples were evaluated in duplicate, in a randomized order and following a Latin square design. The identified attributes were rated on a 5-level scale of perceived intensity from “none” to “a lot”. Table A1 in Appendix A shows the definitions and indications established for the evaluation of the most complex attributes.

### 2.5. Statistical Analysis

Statistical analysis of obtained data was performed with SPSS (IBM, Chicago, IL, USA). Three-way analysis of variance (ANOVA) and Tukey tests were performed for the analysis of fermentation times, post-acidification, TA, bacterial survival, VOCs, and rheology data. Sensory data were analyzed with two-way ANOVA. Statistical evaluation of the results for the intensity perception included a three-way multivariate analysis of variance (MANOVA) with the Wilks test to identify the overall sample differences, and ANOVA to find for which attribute there was significant differences, both considering the factors ”product”, “judge”, and “replicate”, as well as their two-way interactions. The Least Significant Difference (LSD) test was used to detect significant differences among the product samples when attributes had a significant product effect. Rolling correlations were performed with an Excel function with rheological experimental measurements and sensory perception data. Correlation coefficients between 1 and 0.6 and between −1 and −0.6 were accepted as significant. Principal Component Analysis (PCA) was performed to correlate VOCs and flavor attributes for each of the bases and all culture combinations. For all statistical analyses, except for the rolling correlations, significant differences were assumed at 95% confidence intervals (*p* < 0.05).

## 3. Results and Discussion

### 3.1. Effect of LGG^®^ in Acidification Time

The time needed to reach pH 4.5 for all samples was compared to identify differences across bases and a potential effect of LGG^®^ on acidification (Table A2 in Appendix A). There was a significant difference between the fermentation time in the three bases, which was related to the initial pH of the unfermented bases (6.96, 6.16, and 5.96 for soy, oat, and coconut, respectively) and to the different available nutrients for the starter culture. Coconut samples needed shorter times to reach pH 4.5 (from 5.1 to 5.9 h), and oat samples needed longer (6.6 to 8.5 h) than soy samples (6.0 to 7.0 h). Despite the different nutrients, higher buffering capacity should be expected in oat, since it was the base with higher protein content. In fermented soy samples, the three culture combinations containing LGG^®^ needed 35–60 min less to reach pH 4.5 in comparison to those without LGG^®^. In fermented oat samples, fermentation time was also reduced when LGG^®^ was combined with YF-L01, YF-L02, and BY-01. In fermented coconut samples, there was no significant difference between culture combinations and, therefore, LGG^®^ did not affect the fermentation time.

### 3.2. Post-Acidification and Titratable Acidity during Storage at 6 °C

**Figure 1:**
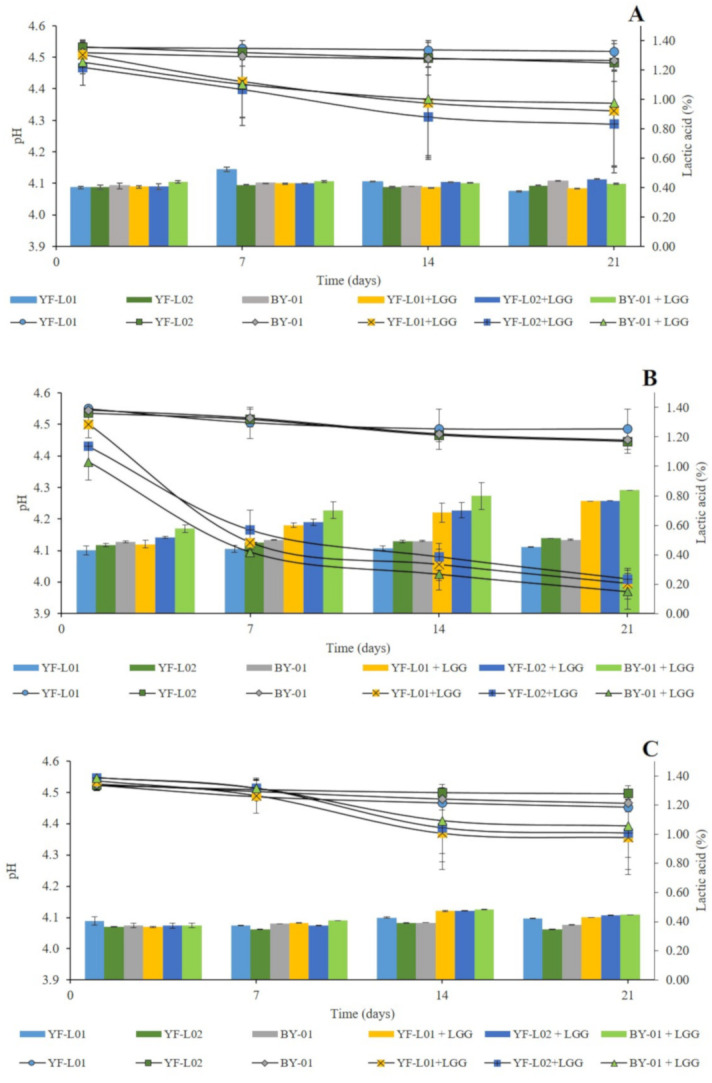
pH (left axis) and titratable acidity (TA) represented as lactic acid percentage (right axis) measurements of fermented (**A**) soy, (**B**) oat, and (**C**) coconut samples at day 1, 7, 14, 21.

Changes in pH and titratable acidity were evaluated in all fermented samples during storage. The analyses were performed at day 1, 7, 14, and 21. Results are shown in Figure 1. Oat samples showed significantly different post-acidification trends in comparison to soy and coconut when LGG^®^ was present. There was no clear difference in acidification patterns in regard to the culture combinations (data not shown). In contrast, in oat samples (Graph B, Figure 1), the presence of LGG^®^ in the culture combination contributed to a more drastic post-acidification and to higher lactic acid levels. These samples reached pH values around 4 after 21 days of storage. This difference can be justified by a higher glucose content in the oat base (Table 3), which could have encouraged LGG^®^ to keep growing and producing lactic acid during storage [54]. This result concurs well with the findings of Helland et al. [55]. They showed the highest lactic acid production by *L. rhamnosus* in comparison to other *Lactobacillus* strains in a plant-based blend. TA of fermented oat in this study was higher than the one reported by Bernat et al. [54]. The highest value was 0.84% in samples fermented by BY-01+LGG^®^ after 21 days of storage. In fermented soy samples, the presence of LGG^®^ showed no significant differences in lactic acid production (Graph A, Figure 1). The highest value was 0.52% in samples fermented with YF-L01 after 7 days of storage. Similar TA values were reported by Mishra et al. in 2019 [56] in fermented soy blends and by Kpodo et al. in fermented soy-peanut blends [57]. In coconut samples (Graph C, Figure 1), LGG^®^ only had an effect at day 14 and 21, when samples showed significantly higher lactic acid content. The highest value was 0.48%, found in oat samples fermented by BY-01+LGG^®^ after 14 days of storage.

### 3.3. Viability of BB-12^®^ and LGG^®^ during Storage at 6 °C

**Figure 2:**
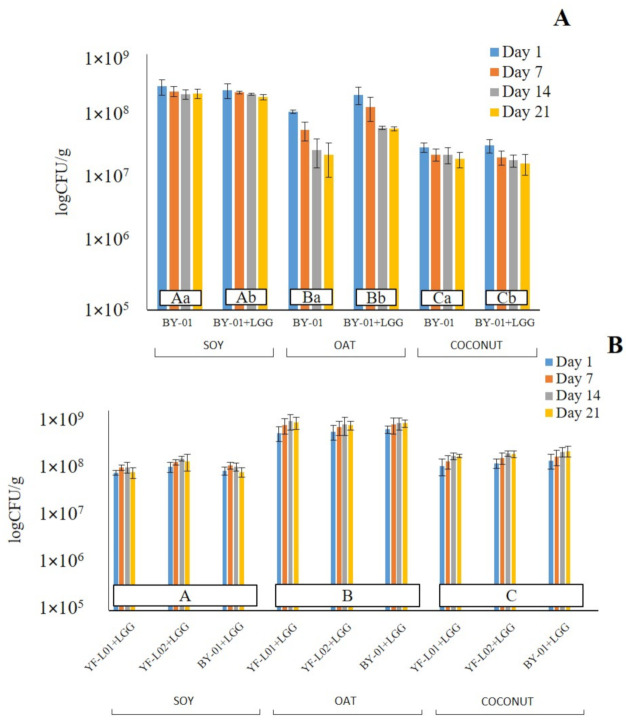
Viable cell counts of (**A**) BB-12^®^ and (**B**) LGG^®^ in fermented soy, oat, and coconut bases in day 1, 7, 14, and 21 of storage at 6 °C. ^ABC^ means with different uppercase superscripts indicate significant differences between different bases (*p* < 0.05). ^ab^ means with different lowercase superscripts indicate significant differences between different culture combinations (*p* < 0.05). Since no significant difference between different cultures in each base was observed in the case of LGG^®^ CFU, only uppercase superscripts are shown.

Viability of BB-12^®^ and LGG^®^ was evaluated through cell count during storage. According to the results shown in Figure 2, all three substrates were suitable for the survival of both probiotic strains. CFU of BB-12^®^ ranged from 1.8 × 10^7^ to 3.2 × 10^8^ CFU/g (Graph A, Figure 2). These values are in line with the results that were obtained by Pavunc et al., who reported BB-12^®^ growth to be higher than 10^6^ CFU/g in fermented cereal matrices [58]. There was a significant interaction between the base and the storage time as well as the culture combination. This indicates that different BB-12^®^ CFU values at different time points or fermented with different cultures depend on the base. The BB-12^®^ cell count was significantly different in the three bases. Highest values were reported in soy, followed by oat, and lastly, coconut. A reasonable explanation for this would be that *Bifidobacterium* was able to metabolize sucrose and GOSs [59], which are present in soy samples, while it was not capable of degrading starch, the main carbon source in oat. The cell count of BB-12^®^ was significantly higher when BY-01 was combined with LGG^®^ only in oat samples. It was also observed that BB-12^®^ viability decreased over storage time in all three bases, but more drastically in oat. In fermented soy and coconut samples, LGG^®^ did not have any significant effect on BB-12^®^ survival.

Regarding the LGG^®^ cell count (Graph B, Figure 2), values ranged from 5.7 × 10^7^ to 6.2 × 10^8^ log CFU/g. These values correlate to the ones previously found in fermented cereal bases [55] and legume bases [39]. The only significant interaction was between the base and the storage time. This indicates that different LGG^®^ CFU at different time points depend on the base. LGG^®^ survival in oat samples was significantly different to soy and coconut. A reasonable explanation may be LGG^®^’s preference for simple carbohydrates [60]. Carbohydrate content in oat was higher than in the other two bases [5]. Thus, LGG^®^ has access to a greater amount of sugars that can be used as a carbon source. YF-L01, YF-L02, and BY-01 did not show any significant difference between them when combined with LGG^®^, which indicates that none of them affected the viability of LGG^®^. The only exception was the soy sample fermented by YF-L02+LGG^®^, whose LGG^®^ CFU were higher in comparison to the other two combinations at day 14 and 21.

All fermented samples showed BB-12^®^ and LGG^®^ CFU values above 10^7^ CFU/g. This would suggest both strains as good candidates for probiotic plant-based fermented products.

### 3.4. Sensory Perception of Fermented Soy, Oat, and Coconut Samples

**Figure 3:**
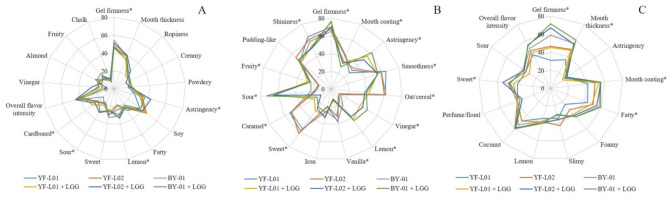
Perception of sensory attributes in (**A**) soy, (**B**) oat, and (**C**) coconut samples fermented with different culture combinations.

The trained panel identified and evaluated different sensory attributes for each of the fermented soy, oat, and coconut samples. Sensory scores for each fermented base are presented in three spider diagrams (Figure 3). Each diagram shows the effect of each culture combination on the perception of each attribute.

Certain attributes were identified and evaluated in all three bases, and therefore, its perception was compared across them. Gel firmness was strongly perceived in oat samples, which could be attributed to a stronger structure formed by gelatinized oat starch, other non-starch polysaccharides, and proteins. This result is supported by the rheology data reported in Section 3.5. Notwithstanding the unidentified effect of LGG^®^ on flow properties, its presence affected sensorial perception of certain textural attributes. Astringency perception was higher in fermented soy and oat samples in comparison to fermented coconut samples. This attribute is related to dryer and rougher raw materials, such as the former ones. Different perception of astringency is attributed to the fat content in coconut samples, which may have smoothened the mouthfeel in comparison to the other two materials. Additionally, polyphenols present in soy and oat could enhance astringency perception. Sweet, lemon, and sour tastes were identified in all three bases, but no significant difference was reported between them. Food materials prone to coalescence enhance the perception of fat in mouth [61], which is supported by the obtained results in this study. Coconut samples scored higher in fatty perception than soy, due to their high fat content. The presence of homogenized fat globules contributes to mouth coating and thickness perception [62]. In fermented soy samples, a potential effect of LGG^®^ on sensory perception of gel firmness, astringency, sourness, lemon taste, and cardboard was observed. Nevertheless, the base contributed to a great extent to the flavor perception, not only in fermented soy, but also in fermented oat and coconut. When it was combined with BY-01, LGG^®^ increased sourness perception and decreased gel firmness perception in fermented soy samples. In combination with YF-L02, astringency, sourness, and lemon taste increased. In fermented oat samples, gel firmness, astringency, vinegar, lemon taste, fruity taste, and sourness perception was higher when LGG^®^ was present in the culture combination. Some of these attributes are linked to acid foods, and, although the acid content of LGG^®^-containing oat samples could not be used for the statistical analyses, the results of the titratable acidity experiment indicated that these samples contained higher lactic acid content. This supports the hypothesis that LGG^®^ could have enhanced acidification. In contrast, mouth coating, smoothness, shininess, sweetness, oat/cereal, vanilla, and caramel taste perception were lower in these samples. In coconut samples, perception of gel firmness, mouth coating, mouth thickness, sweetness, and fatness showed significant differences across samples. All samples containing LGG^®^ were perceived as firmer in comparison to the ones fermented without LGG^®^. In combination with YF-L01, LGG^®^ increased mouth thickness and mouth coating. Perfume odor was specifically evaluated in coconut samples. Esterification of free fatty acids in coconut with ethanol, which was higher than in soy and oat samples, may have generated ethyl esters with floral odors [34]. However, no significant difference was found in its perception across the different samples.

### 3.5. Rheological Behavior

**Figure 4:**
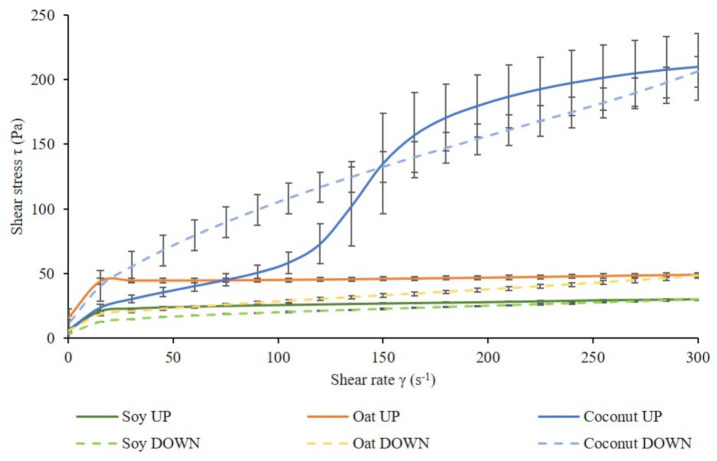
Flow curves under controlled shear rates from 0 to 300 s^−1^ and from 300 to 0 s^−1^ of fermented soy, oat, and coconut samples. Continuous lines correspond to up-flow and discontinuous lines to down-flow.

Figure 4 shows the flow curves of fermented soy, oat, and coconut samples. The shape of all flow curves was the same among different culture combinations. Therefore, one flow curve per fermented base was plotted.

The literature supports that fermented soy-based beverages [63], oat-based preparations [1], and coconut yogurts [64] have a shear thinning behavior. Nevertheless, the flow test revealed shear thinning in fermented soy and oat samples, but unexpected behavior in fermented coconut samples. The microstructure in each of the plant bases was expected to be different from each other, due to the very different matrix components. Therefore, different flow behavior and different shear stress values were expected. An hysteresis loop, typical of dairy fermented products, was observed in fermented soy and oat samples. In contrast, the fermented coconut up-flow curve had an atypical S-shape and significantly higher shear stress values in comparison to the other two bases. Fat crystals may have formed in these fermented samples due to the high fat content of coconut milk [65]. These crystals form a three-dimensional structure that confer resistance to deformation [66]. Shear thickening behavior was observed in fermented coconut samples up to 100 s^−1^, which could be attributed to a dominating starch structure. At higher shear rates, shear thinning behavior was observed, indicating a potential breakdown of the starch.

Shear stress values of fermented soy, oat, and coconut at a shear rate of 45.2 s^−1^ were evaluated (Table 4). There was no significant interaction between the effect of the base and the effect of the culture in fermented soy and oat samples. No significant differences between the culture combinations were found in any of the fermented bases (data not shown), but, as expected, all fermented bases were significantly different from each other.

Oat shear stress values at a 45 s^−1^ shear rate were significantly higher than those in soy and coconut. A reasonable explanation could be the higher protein content in oats in comparison to soy and coconut, in combination with their starch, which may be responsible for a higher viscosity. A firmer structure could have been formed from β-glucans already present in oat [45]. β-glucans have a high water-holding capacity, which could have enhanced gel strength. Their gelling properties can increase viscosity in liquid solutions [67]. β-glucans can form a polysaccharide-protein matrix where fat droplets are held [67]. Another type of polysaccharide that contributes to improvement of the textural and rheological properties of fermented foods is EPS. Previous studies suggested the contribution of EPS to gel firmness in fermented products [68], and Martensson et al. observed EPS produced by *L. delbrueckii* subsp. *bulgaricus* in oats [43]. EPS production has not been determined in each plant base; therefore, the culture combination could have been contributed in a different way in each of them. Soy protein can aggregate at its isoelectric point (pH 4.5) [69], forming a gel network that contains other matrix components. Soy samples in this study showed lower shear stress and G* values than the other two bases and, therefore, lower viscosity. This indicates weaker gel structures in fermented soy samples. Grygorczyk reported that the composition and processing of soy proteins may affect gelation properties [62]. The different culture combinations did not have any significant effect in any of the bases. These results indicate that the addition of LGG^®^ did not have any negative effect in the flow behavior of any of the samples.

The hysteresis area of fermented soy and oat samples represents the structural recovery during shear, and it is shown in Table 5. It was not possible to calculate the hysteresis area for coconut samples, since the obtained loop was not regular (Figure 4).

Soy samples fermented with YF-L01+LGG^®^ showed higher hysteresis area values than those fermented with YF-L01. Oat samples did not show any significant differences between culture combinations. Therefore, LGG^®^ did not have any effect on their structural recovery.

**Figure 5:**
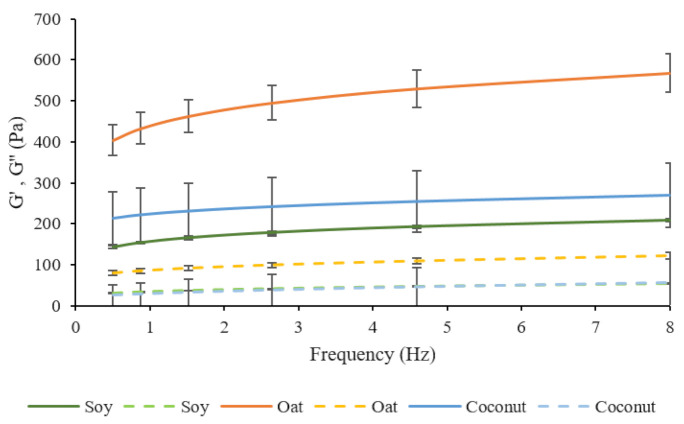
Dynamic mechanical spectra in frequency sweeps of fermented soy, oat, and coconut samples. Continuous lines correspond to G’, discontinuous lines correspond to G”.

An oscillation test was performed to evaluate the viscoelastic properties of fermented soy, oat, and coconut samples. Storage modulus (G’) and loss modulus (G”) determine the elastic and viscous components of all fermented bases, respectively, and are shown in Figure 5. All samples showed higher G’ values than G” for all culture combinations, revealing a gel-like rheological behavior. Donkor et al. reported similar G’ values for fermented soy milk with probiotic cultures [15]. Brückner–Gühman et al. stated that, in fermented oat samples, protein aggregates, starch granules, and fat droplets increase the rigidity of the gel [70]. This could explain high G’ values in fermented oat samples in this study. They also concluded that the main contributor to the stability of an oat concentrate gel would be the swollen starch, instead of an oat protein network. G* indicates the firmness of the samples and it was chosen to compare all three bases (Table 4). G* values of fermented soy, oat, and coconut were significantly different from each other, and oat samples showed a higher complex modulus. Different culture combinations did not have any significant effect in G* in any of the bases, which showed no significant effect of LGG^®^ in complex modulus.

#### 3.5.1. Correlation between Instrumental Measurements and Texture Perception

A combination of sensory perception and physical measurements allows a more precise characterization of food materials. Therefore, up-flow shear stress and complex modulus were correlated with evaluated textural attributes in each sample (Table 6 and Table 7). The correlations were different across bases, which reflects different microstructures in each fermented base. In soy samples, gel firmness was positively correlated to shear stress and to G*. Mouth thickness was positively correlated to shear stress from 90.2 s^−1^ onwards. Shear stress in fermented oat samples was positively correlated with gel firmness along the entire flow curve and with mouth coating and smoothness below 120 and 75.2 s^−1^, respectively. This is supported by the findings of Buldo et al. [68]. Nevertheless, G* of fermented oat samples was positively correlated with gel firmness, but negatively correlated with mouth coating and smoothness perception. In coconut samples, gel firmness, mouth thickness, and mouth coating showed positive correlation with shear stress and G* at all shear rates and frequencies, respectively. Fat crystals increase the elastic modulus and increase firmness perception [9]. Consequently, when shear stress and G* increase, the perception of these attributes will be enhanced. These results encourage further investigation on the rheological behavior of fermented plant-bases and its effect on sensory perception.

### 3.6. Volatile Organic Compounds

To evaluate the effect of fermentation with and without LGG^®^ on VOCs, the obtained results were classified in two categories: major contributors to fermented dairy flavor, and characteristic VOCs of each fermented raw material. This was done to observe the potential increase of dairy flavor and reduction of off-flavors.

#### 3.6.1. Major Contributors to Fermented Dairy Flavor

Fermented soy, oat, and coconut bases were screened for targeted VOCs by GC-FID, and results are shown in Table 8. When all fermented bases were compared, diacetyl, acetoin, acetaldehyde, acetone, and ethanol levels were significantly different in all three bases. Ethyl acetate, an ester that is found in lactobacilli fermented products [71] and in dairy cheese [72], was targeted but not identified in any of them by GC-FID.

Diacetyl, acetoin, and acetaldehyde play a key role in dairy flavor perception [73] and are products of pyruvate degradation. Pyruvate can be metabolized through two main pathways. The first one converts pyruvate into α-acetolactate, which is decarboxylated to diacetyl in aerobic conditions without any enzymatic activity. Diacetyl can then be reduced to acetoin, and acetoin to 2-3-butanediol. The second one transforms pyruvate into acetaldehyde through acetyl-coenzyme A (acetyl-CoA) [74]. The effect LGG^®^ can cause on the levels of these pyruvate breakdown products can be relevant for the sensory perception of fermented samples. Acetoin and diacetyl provide buttery odors that are responsible for caramel and sweet flavors, respectively [34]. Acetoin is generally produced in higher amounts than diacetyl through citrate metabolism, which was reflected in all three fermented bases (Table 8). Both compounds were significantly higher in fermented oat samples, followed by coconut and soy. This could be explained by a higher fermentable carbohydrate content in oat samples. LGG^®^-containing samples reflected higher acetoin levels in all three bases in comparison to samples that were fermented with culture combinations without LGG^®^. Consequently, it could be assumed that LGG^®^ has high diacetyl-reductase activity to transform diacetyl into acetoin compared to the starter cultures. Acetoin was produced in lower quantities in soy samples by BY-01+LGG^®^ and YF-L01+LGG^®^ in comparison to YF-L02+LGG^®^. LGG^®^ increased diacetyl levels in oat and coconut when it was combined with YF-L01 and BY-01. In contrast, LGG^®^ decreased them in combination with YF-L02 in all three bases. Kaneko et al. reported a relationship between excessive amounts of diacetyl and unpleasant odors [74]. This suggests that YF-L02 without LGG^®^ would not be an appropriate candidate to ferment the oat matrix investigated in this study due to the high diacetyl levels that were identified in this sample. One of the main principal components of fermentation aroma in yogurt is acetaldehyde [74]. Samples that were fermented with LGG^®^-containing culture combinations showed significantly lower acetaldehyde content in all three bases. This could be due to a low ability of *L. rhamnosus* to produce it from this specific matrix, or due to a high enzymatic activity to convert it into ethanol. Acetaldehyde is a product of pyruvate degradation by acetyl-coenzyme A, but it can also be produced through other metabolic pathways from citrate by *S. thermophilus* (strain present in YF-L01, YF-L02, and BY-01). Acetone content was significantly higher in soy samples. It is a compound that confers apple and solvent flavor and has been identified in dairy products, such as kefir [71]. Previous studies reported its presence in fermented soy and considered it as another main contributor to the flavor and aroma in yogurt [75]. However, LGG^®^ did not show any significant effect on its production in any of the three bases. Ethanol levels were significantly higher in coconut samples, but different culture combinations did not have a significant effect on its content. In contrast, LGG^®^ significantly reduced ethanol levels in fermented oat samples when it was combined with YF-L01, but increased them when it was combined with BY-01. Kpodo et al. detected ethanol in their fermented matrix containing peanut and soy, but not in their dairy yogurt control [57]. They attributed its presence to glucose breakdown and amino acid catabolism. Nevertheless, they remarked that it is not a relevant contributor to dairy flavor profiles, but probably a complementary one. Ethanol can also react with free fatty acids and be further converted into ethyl esters, which would provide floral and fruity odors [34].

3-methyl-butanal was found in all fermented oat samples and in nonsignificant amounts in the other two fermented bases. Yan Chun et al. identified it in soy milk, although it had a low flavor dilution factor and, therefore, was not considered a major flavor component [76]. This compound derives from an enzymatic reaction of leucine [77] and has been already identified in oat samples by Salmenkallio–Marttila [78] and by Lee et al. [79] as a product of amino acid degradation by *L. paracasei*. Natrella et al. characterized it as the most relevant volatile compound in mozzarella cheese, providing a nutty and fresh cheese odor [80]. Dan et al. reported higher 3-methyl-butanal content when dairy milk was fermented by a mixed culture of *L. bulgaricus* and *S. thermophilus* when the rates of the latter one were higher [81]. This supports the results of this study, where oat samples fermented with YF-L01 without LGG^®^ (pure *S. thermophilus*) reflected higher levels of 3-methyl-butanal than the one fermented with YF-L01+LGG^®^. 1-Butanol was not identified in sufficiently high amounts, and therefore it was not reported in Table 8.

#### 3.6.2. Characteristic VOCs of Soy, Oat, and Coconut (Reported as Signal-to-Noise)

Fifty-four untargeted volatile compounds were identified by GC-MS in all three fermented bases (data not shown). They comprised ketones, esters, acids, aldehydes, alcohol, furans, sulfurs, lactones, terpenes, benzenes, and aromatic compounds. Twelve of them (ethyl acetate, 2,3-pentanedione, hexanal, ethyl decanoate, α-pinene, benzaldehyde, 3-carene, acetoin, δ-decalactone, γ-octalactone, dimethyl disulfide, and 2-heptanone) were present in the three fermented bases. Previous studies were taken as references for predominant volatiles of soy [76,82,83], oat [27,79], and coconut [84,85]. Compounds identified in this study (Table A3–Table A5 in Appendix C) were compared to their results. Two compounds were present in all fermented samples, namely, hexanal and benzaldehyde. Hexanal is mainly produced through linoleic acid oxidation, and it can be further oxidized to hexanoic acid and reduced to 1-hexanol by dehydrogenase enzymes during fermentation processes [79]. Since linoleic acid is the primary fatty acid in oat [79] and soybeans [86], detection of hexanal in fermented soy and oat samples was expected in this study. Hexanal content was significantly higher in these two fermented bases in comparison to fermented coconut samples, where it was found in very low quantities, as previous studies suggested [85]. Achouri et al. detected its presence in soy milk and in soy blends and observed changes in its levels during storage, attributing them to further lipid oxidation [82]. Hexanal is associated with green and beany odors [83], but also related to rancidity [28]. Nevertheless, Sides et al. stated that, since hexanal is present in oat samples with acceptable flavors, perception of rancidity is not directly linked to the presence of hexanal, but to its concentration [87]. Regarding benzaldehyde, a compound found in Camembert cheese [88], it was significantly different in all three bases and higher in fermented oat samples, followed by coconut and soy samples. Achouri et al. and Kaczmarska et al. reported its presence in soy milk products and in germinated soy, but stated its minor contribution to the soybean aroma [82,83]. Salmenkallio [78], McGorrin [27], and Lee [79] detected benzaldehyde in uncooked and cooked oatmeal and attributed it to an almond odor. Its presence could be associated with reducing sugars and amino acids interactions [87]. Wang et al. identified benzaldehyde in coconut milk [89], and fermented coconut samples in this study were the only ones showing significantly different benzaldehyde content between the different culture combinations (Table 8). However, LGG^®^ had no significant effect on its production.

The main contributors to soy flavor (in addition to hexanal and benzaldehyde) are nonanal, heptanal, octanal, acetic acid, 1-hexanol, 1-pentanol, 1-octen-3-ol, heptanol, 2-pentyl furan, 2-ethyl furan, 1-octen-3-one [76,82,83]. From this list, only hexanal, 2-pentyl furan and benzaldehyde were identified in fermented soy samples in this study, and none of the different culture combinations had an effect on their quantity. Nevertheless, other volatile compounds were found in fermented soy samples, and those whose production was affected by LGG^®^ are shown in Table A3 in Appendix C. The sample fermented with YF-L01 was not included due to a lack of reliable replicates for the statistical analysis of VOCs. 3-Methyl-butanal, dimethyl trisulfide, 2-undecenal, and δ-dodecalactone were identified with values under the level of detection, and therefore, it was not possible to properly quantify their presence. γ-Nonalactone was only detected in samples fermented with BY-L01+LGG^®^ at a signal-to-noise value of 12. 2,3-Pentanedione is considered a dairy yogurt flavor contributor [90] and was detected in high levels in fermented soy samples, in line with the findings of Kaneko et al. [74] and Ahmad et al. [91]. A combination of LGG^®^ with BY-01 significantly increased the levels of this compound. Ethyl octanoate and ethyl decanoate were detected in all fermented soy samples, but the former was under the levels of detection in the sample fermented with BY-01. A combination of LGG^®^ with YF-L02 significantly decreased their production, but it was significantly enhanced when combined with BY-01. Ahmad et al. found both compounds in cheddar cheese, but only the former in soy cheese [91]. They also identified lactones in dairy cheese, but not in soy cheese. In contrast, soy samples in this study fermented with YF-L02 and BY-01+LGG^®^ contained remarkable levels of δ-decalactone and δ-octalactone. This would be a positive aspect of soy fermentation with the culture combinations used in this study for dairy alternatives. However, there was no significant effect of the cultures on their production, and it was not possible to quantify lactones in other fermented samples because the values were between levels of detection and levels of quantification. Regarding terpenes, there was a significant decreasing effect of LGG^®^ in limonene levels when combined with YF-L02. Moreover, α-pinene levels decreased when LGG^®^ was combined with YF-L02, but increased in combination with BY-01. In the case of hexanal, its content was significantly decreased when LGG^®^ was combined with YF-L01, but significantly increased in combination with BY-01. The lowest levels of this compound were found in samples fermented with BY-01 and with YF-L01+LGG^®^, which would suggest these combinations as appropriate to remove one of the main responsible compounds for beany flavor [19]. 2-pentyl-furan, a product of the degradation of fatty acids, also contributes to soy flavor, and was found in high amounts in all fermented samples, but significantly decreased when LGG^®^ was combined with YF-L02.

According to McGorrin [27] and Lee et al. [79], the key volatile compounds of oat flavor are hexanal, nonanal, benzaldehyde, 3- methyl-butanal, octanal, 1-hexanol, 1-pentanol, 1-octen-3-ol, 3-methyl-1-butanol, 2-ethylfuran, 2- heptanone, 3-hydroxy-2-butanone, and 3,5-octadien-2-one. However, only hexanal, benzaldehyde, 3-methyl-butanal, octanal, 2-ethyl-furan, and 1-hexanol were found in fermented oat samples of this study. Previous literature reported variations in volatile compounds with fermentation time in oats [79]. Initial fermentation stages were associated with aldehydes and later stages with acids, alcohols, ketones, and furans. Table A4 in Appendix C shows VOCs in whose content LGG^®^ had a significant effect. 2-3-Pentanedione was previously identified in oats [92], and it is one of the main flavor components of fermented dairy milk [93]. Its presence in fermented oat samples in higher levels than in fermented soy and coconut samples may suggest oat’s potential for fermented dairy alternatives. However, 2-3-Pentanedione levels decreased when the culture combination contained LGG^®^. Although previous studies did not find ethyl acetate in dairy yogurt, Beshkova et al. detected its presence in kefir [71] and it was also detected by GC-MS in the fermented oat samples in this study. Lee et al. identified nonanal, hexanal, 2-pentylfuran, 1-octen-3-ol, and 2-nonenal as lipid degradation products [79]. They observed that hexanal content decreased during fermentation of oats, while 1-hexanol levels increased. This could explain the presence of the latter compound in fermented oat samples. Toluene, ethanol, and 2-propanol were the VOCs that were found in higher amounts in fermented oat. Toluene did not vary among culture combinations and, therefore, was not reported in the table, but it has been found in dairy yogurts [90] and also in fermented oats [94]. In regard to alcohol levels, 2-propanol drastically decreased when YF-L01 was combined with LGG^®^. In samples where YF-L02 was supplemented with LGG^®^, 2-3-pentanedione levels increased and limonene levels decreased. The combination of BY-01 with LGG^®^ increased ethyl acetate content, but decreased the amount of 2-3-pentanedione and furfural.

The predominant compounds of coconut flavor according to the literature [84,85] are hexanal, 2-heptanone, nonanal, acetic acid, 2-ethylfuran, 2-pentanone, ethyl lactate, ethyl acetate, δ-octalactone, δ-decalactone, dodecanoic acid, octanoic acid, 1-hexanol, phenyl ethyl alcohol, 2-methyl tetrahydrofuran, tetradecanone, hexadecanone, ethyl octanoate, ethyl acetate, and ethyl decanoate. From all of them, only the first 10 compounds were identified in fermented coconut samples in this study. Ethyl lactate, previously characterized as the main flavor component in coconut variety *neera* [84], was detected in all samples. Furthermore, phenylacetaldehyde was also found in all samples at low levels. It is a derivative compound from phenyl ethyl alcohol, another key aroma contributor in coconut [84], which would explain its presence. δ-octalactone and δ-decalactone were also present in all fermented coconut samples, as well as butyrolactone. This was expected since they are major contributors to the coconut flavor derived from hydroxy acids [95]. Table A5 in Appendix B shows untargeted VOCs in fermented coconut samples whose content was affected by LGG^®^. LGG^®^ increased the content of 2-pentanone and decreased the content of 2-3-pentanedione when it was combined with YF-L01. In combination with YF-L02, LGG^®^ increased 2-pentanone and α-pinene production, but decreased that of 2-3-pentanedione. BY-01 supplemented with LGG^®^ increased 2-pentanone, γ-nonalactone, and butyrolactone levels, but decreased 2-3-pentanedione content.

#### 3.6.3. Correlation between Instrumental Measurements and Flavor Perception

**Figure 6:**
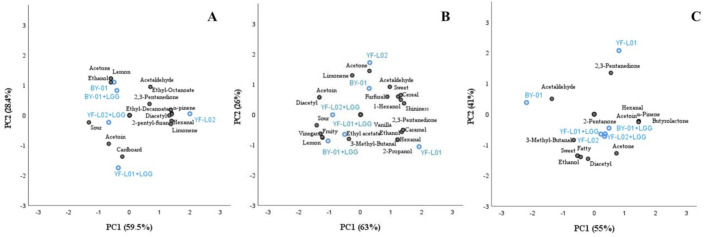
Effect of different culture combinations and the volatile organic compounds they produced on the sensory perception of flavor attributes. PCA bi-plot of the first two principal components (PCs) in fermented (**A**) soy, (**B**) oat, and (**C**) coconut samples labeled by culture combinations.

PCA correlated VOCs and perception of sensorial attributes identified in each fermented sample (Figure 6). VOCs and flavor attributes showing significant differences between culture combinations in each base were selected for the analysis. Hexanal was included in all three analyses due to its contribution to off-flavors in plant-based products [83].

PCA of fermented soy samples explains 87.9% of the total variance, comprising 59.5% of the first PC and 28.4% of the second PC. YF-L01 was not included in the PCA of soy samples due to a lack of reliable replicates in VOCs analysis, as mentioned above. Samples fermented with YF-L02 were far from those fermented with YF-L02+LGG^®^ along PC2 axis. In contrast, BY-01 and BY-01+LGG^®^ were located remarkably close to each other. Therefore, an effect of LGG^®^ when combined with YF-L02, but not when combined with BY-01 was observed in soy samples. Perception of sourness was negatively correlated (R = −0.9) with α-pinene, 2-pentyl-furan, hexanal, diacetyl, ethyl-decanoate, ethyl-octanoate, and limonene. Simultaneously, α-pinene, 2-pentyl-furan, hexanal, and diacetyl were positively correlated (R = 0.9) with ethyl-decanoate, ethyl-octanoate, and limonene. Lemon flavor was better perceived when acetone and ethanol were produced (R = 0.9). BY-01 and BY-01+LGG^®^ clustered together around these components, which would point at a contribution of BY-01 and not LGG^®^ to their production. PCA of fermented oat samples explained 89% of the total variance, comprising 63% of the first PC and 26% of the second PC. All culture combinations containing LGG^®^ clustered together far from their respective cultures without LGG^®^, which indicated that LGG^®^ had an effect on fermented oat flavor. They were close to the attribute sourness and to lemon and fruity flavors. A positive correlation between them and the presence of diacetyl and acetoin (R = 0.9–1) was found. Sweetness and cereal flavor were related with the presence of acetaldehyde (R = 0.7) and acetone (R = 0.4–0.5), although the association was not significantly strong. Samples including LGG^®^ in the culture were plotted far from these components, reflecting higher acidification. YF-L01 was found close to ethanol and 2-propanol, and these compounds were negatively correlated to diacetyl and acetoin (R = −0.9), which is supported by the results obtained by GC-FID. PCA of fermented coconut samples explained 96% of the total variance, comprising 55% of the first PC and 41% of the second PC. Sweetness and fattiness were positively correlated with ethanol (R = 0.9) and diacetyl (R = 1), but negatively correlated with the presence of 2,3-pentanedione (R = 9). The presence of acetoin was positively correlated (R = 1) with δ-nonalactone, butyrolactone, α-pinene, and 2-pentanone. The sample fermented with YF-L02 clustered with all culture combinations containing LGG^®^ around the previously mentioned attributes. This indicates that LGG^®^ did not have any effect on fermented coconut flavor when added to YF-L02. Samples fermented with BY-01 were associated with acetaldehyde, which is supported by the results obtained by GC-FID.

## 4. Conclusions

By combining LGG^®^ with the studied starter cultures, significant effects in fermented plant bases were reported. Acidification time was improved with its presence in fermented soy and oat samples. LGG^®^ and BB-12^®^ were able to grow and survive in all three fermented bases. LGG^®^ did not have any negative impact on the rheological behavior of the fermented bases. However, it was shown that it was different in all three bases. This reflects an important role of the base on the texture. Supplementation with LGG^®^ resulted in higher acetoin levels and lower acetaldehyde levels in all three bases. Diacetyl content was also enhanced in oat and coconut samples when LGG^®^ was combined with YF-L01 and BY-01. Regarding sensory perception of fermented samples, major effects of LGG^®^ were observed in oat samples. LGG^®^ increased the perception of acid-related flavor attributes and decreased the sweetness and oat/cereal taste. LGG^®^ increased the gel firmness perception in the fermented coconut samples. On this basis, the results of this study encourage future research on the potential of probiotic LAB for the improvement of physicochemical properties in plant-based products.

## Figures and Tables

**Table 1:** Composition of the plant bases.

Base	Composition
Soy	95% soy milk, 5% sucrose
Oat	30% *w*/*w* oat concentrate
Coconut	93% coconut milk, 3% sucrose, 4% starch

**Table 2:** List of bacterial cultures used for the fermentation of the plant bases.

Culture Name	Composition
YOFLEX^®^* YF-L01 DA	*Streptococcus thermophilus*
YOFLEX^®^* YF-L02 DA	*Streptococcus thermophilus* and *Lactobacillus bulgaricus* supplemented with
	*Lactobacillus acidophilus*, *Lactobacillus paracasei*, and *Bifidobacterium*
NU-TRISH^®^* BY-01 DA	*Streptococcus thermophilus* and *Lactobacillus bulgaricus* with *Bifidobacterium*, BB-12^®^
LGG^®^	*Lactobacillus rhamnosus*

YOFLEX^®^ and NU-TRISH^®^ are trademarks of Chr. Hansen A/S.

**Table 3:** Nutritional content of the unfermented bases produced for this study.

Base	Protein (%)	Carbohydrates (%)	Main Sugar	Fat (%)
Soy	3.7	5	Sucrose	2
Oat	4.5	18	Glucose	2.2
Coconut	1.49	3	Sucrose	17.67

**Table 4:** Shear stress (τ) and complex modulus (G*) of fermented soy, oat, and coconut samples at a controlled shear rate of 42.5 s^−1^ and an oscillatory frequency of 1.52 Hz, respectively.

Base	τ (Pa)	G* (Pa)
Soy	24 ^A^	170 ^A^
Oat	45 ^C^	472 ^C^
Coconut	36 ^B^	233 ^B^

^ABC^ Means with different uppercase superscripts in the same column indicate significant differences between different bases (*p* < 0.05).

**Table 5:** Hysteresis loop area of fermented soy and oat samples.

Culture Combination	Base	
	Soy	Oat
YF-L01	1240 ^a^	3849
YF-L02	1286 ^ab^	3694
BY-01	1276 ^ab^	4505
YF-L01+LGG^®^	1357 ^b^	4020
YF-L02+LGG^®^	1328 ^ab^	4016
BY-01+LGG^®^	1346 ^ab^	3987

^ab^ Means in the same column with different lowercase superscripts indicate significant differences between different culture combinations (*p* < 0.05).

**Table 6:** Correlation coefficient between up-flow and textural attributes identified in each fermented base.

Shear Rate (s^−1^)	SOY				OAT			Coconut		
	Textural Attributes				Textural Attributes			Textural Attributes		
	Gel Firmness	Mouth Thickness	Ropiness	Creaminess	Gel Firmness	Mouth Coating	Smoothness	Gel Firmness	Mouth Thickness	Mouth Coating
0.3	0.0	−0.3	0.0	0.4	0.9 *	−0.3	−0.4	0.8 *	0.8 *	0.7 *
15.3	0.9 *	0.4	−0.5	0.1	1.0 *	−0.7 *	−0.7 *	0.9 *	0.8 *	0.8 *
30.2	0.6 *	0.5	−0.5	0.3	1.0 *	−0.6 *	−0.6 *	0.9 *	0.8 *	0.8 *
45.2	0.6 *	0.4	−0.5	0.4	1.0 *	−0.6 *	−0.6 *	0.8 *	0.8 *	0.8 *
60.2	0.6 *	0.5	−0.5	0.3	1.0 *	−0.7 *	−0.6 *	0.8 *	0.8 *	0.7 *
75.2	0.5	0.5	−0.5	0.3	1.0 *	−0.6 *	−0.6 *	0.8 *	0.8 *	0.7 *
90.2	0.6 *	0.6 *	−0.5	0.3	0.9 *	−0.6 *	−0.5	0.8 *	0.8 *	0.7 *
105.0	0.6 *	0.6 *	−0.5	0.3	0.9 *	−0.6 *	−0.5	0.8 *	0.8 *	0.7 *
120.0	0.5	0.6 *	−0.5	0.3	0.9 *	−0.6 *	−0.5	0.8 *	0.8 *	0.6 *
135.0	0.5	0.6 *	−0.5	0.3	0.9 *	−0.5	−0.4	0.8 *	0.8 *	0.6 *
150.0	0.5	0.6 *	−0.5	0.3	0.9 *	−0.5	−0.4	0.8 *	0.8 *	0.6 *
165.0	0.5	0.6 *	−0.4	0.3	0.9 *	−0.5	−0.4	0.8 *	0.8 *	0.6 *
180.0	0.6 *	0.6 *	−0.5	0.3	0.9 *	−0.5	−0.4	0.8 *	0.8 *	0.7 *
195.0	0.5	0.6 *	−0.5	0.3	0.9 *	−0.4	−0.3	0.8 *	0.8 *	0.8 *
210.0	0.5	0.6 *	−0.5	0.3	0.8 *	−0.4	−0.3	0.8 *	0.9 *	0.8 *
225.0	0.5	0.6 *	−0.4	0.3	0.8 *	−0.4	−0.3	0.8 *	0.9 *	0.8 *
240.0	0.5	0.7 *	−0.4	0.2	0.7 *	−0.4	−0.2	0.8 *	0.9 *	0.8 *
255.0	0.6 *	0.7 *	−0.4	0.2	0.8 *	−0.3	−0.2	0.9 *	0.9 *	0.8 *
270.0	0.6 *	0.6 *	−0.4	0.2	0.8 *	−0.3	−0.2	0.9 *	0.9 *	0.8 *
285.0	0.6 *	0.6 *	−0.5	0.2	0.8 *	−0.3	−0.2	0.9 *	0.9 *	0.8 *
300.0	0.6 *	0.6 *	−0.5	0.2	0.8 *	−0.3	−0.1	0.9 *	0.9 *	0.8 *

* Asterisk denotes significant correlation between rheological parameter and textural attribute (value between 0.6–1 and (−0.6)–(−1)).

**Table 7:** Correlation coefficients between G* and textural attributes identified in each fermented base.

Frequenzy (Hz)	SOY				OAT			Coconut		
	Textural Attributes				Textural Attributes			Textural Attributes		
	Gel Firmness	Mouth Thickness	Ropiness	Creaminess	Gel Firmness	Mouth Coating	Smoothness	Gel Firmness	Mouth Thickness	Mouth Coating
0.5	0.8 *	0.4	−0.5	0.3	0.9 *	−0.7 *	−0.7 *	0.9 *	0.8 *	0.9 *
0.9	0.8 *	0.3	−0.5	0.3	0.9 *	−0.7 *	−0.7 *	0.9 *	0.8 *	0.9 *
1.5	0.8 *	0.3	−0.5	0.3	0.9 *	−0.7 *	−0.7 *	0.9 *	0.8 *	0.9 *
2.6	0.9 *	0.3	−0.5	0.3	0.9 *	−0.8 *	−0.7 *	0.9 *	0.8 *	0.9 *
4.6	0.9 *	0.2	−0.5	0.3	0.9 *	−0.8 *	−0.7 *	0.9 *	0.8 *	0.9 *
8.0	0.8 *	0.0	−0.4	0.3	0.9 *	−0.8 *	−0.8 *	0.9 *	0.8 *	0.9 *

* Asterisk denotes significant correlation between rheological parameter and textural attribute (value between 0.6–1 and (−0.6)–(−1)).

**Table 8:** Targeted Volatile Organic Compounds (VOCs) identified by GC-FID in fermented soy, oat, and coconut samples.

Base	Culture Combination	Acetaldehyde	Diacetyl	Acetoin	Acetone	3-Methyl-Butanal	Ethanol
Soy	YF-L01	2.7 ^Ade^	4.0 ^Ab^	15 ^Abc^	1.5 ^Aab^	n/s	0.7 ^Aa^
	YF-L02	2.5 ^Ad^	7.0 ^Ac^	14 ^Aab^	1.5 ^Aab^	n/s	2.0 ^Aab^
	BY-01	2.9 ^Ae^	3.6 ^Aab^	9.4 ^Aa^	1.6 ^Ab^	n/s	4.1 ^Ac^
	YF-L01 + LGG^®^	1.2 ^Ab^	3.2 ^Aab^	20 ^Ac^	1.3 ^Aa^	n/s	1.2 ^Aa^
	YF-L02 + LGG^®^	0.6 ^Aa^	3.9 ^Aab^	26 ^Ad^	1.6 ^Ab^	n/s	2.6 ^Ab^
	BY-01 + LGG^®^	1.8 ^Ac^	3.0 ^Aa^	19 ^Ac^	1.7 ^Ab^	n/s	5.6 ^Ad^
Oat	YF-L01	1.1 ^Bb^	4.3 ^Ba^	40 ^Ba^	0.3 ^B^	1.2 ^c^	93 ^Bd^
	YF-L02	1.1 ^Bb^	39 ^Be^	42 ^Ba^	0.4 ^B^	0.2 ^ab^	17 ^Bb^
	BY-01	2.5 ^Bc^	8.3 ^Bb^	42 ^Ba^	0.4 ^B^	0.1 ^ab^	8.2 ^Ba^
	YF-L01 + LGG^®^	0.3 ^Ba^	17 ^Bcd^	98 ^Bc^	0.3 ^B^	0.3 ^b^	25 ^Bc^
	YF-L02 + LGG^®^	0.2 ^Ba^	18 ^Bd^	81 ^Bb^	0.3 ^B^	0.2 ^ab^	18 ^Bbc^
	BY-01 + LGG^®^	0.3 ^Ba^	16 ^Bc^	87 ^Bb^	0.3 ^B^	0.1 ^a^	17 ^Bb^
Coconut	YF-L01	3.4 ^Ca^	3.3 ^Ca^	51 ^Cb^	0.4 ^C^	n/s	71 ^C^
	YF-L02	3.1 ^Cb^	18 ^Cd^	48 ^Cab^	0.4 ^C^	n/s	72 ^C^
	BY-01	3.9 ^Cc^	3.6 ^Ca^	40 ^Ca^	0.4 ^C^	n/s	72 ^C^
	YF-L01 + LGG^®^	2.0 ^Cd^	14 ^Cb^	68 ^Cc^	0.5 ^C^	n/s	74 ^C^
	YF-L02 + LGG^®^	0.6 ^Ce^	16 ^Cc^	72 ^Cc^	0.6 ^C^	n/s	72 ^C^
	BY-01 + LGG^®^	0.4 ^Cf^	13 ^Cb^	69 ^Cc^	0.6 ^C^	n/s	73 ^C^

^ABC^ Means in the same column with different uppercase superscripts indicate significant differences between different bases (*p* < 0.05). ^abcdef^ Means in the same column inside each base with different lowercase superscripts indicate significant differences between different culture combinations (*p* < 0.05). “n/s” stands for “no signal”. Contents are measured in parts per million.

## Data Availability

The data presented in this study are property of Chr. Hansen A/S.

## Appendix A. List of Attributes Evaluated during the Sensory Analysis

**Table A1:** Description of analyzed sensory attributes.

Attribute	Definition	Indications
Gel firmness	Resistance to deformation of the product.	Slowly take a spoon of the product and place it on the untouched sample surface. Note how long it keeps its shape.
Ropiness	Sticky, glutinous or soft nature of the product.	Dip the bottom of the spoon several times fast in the surface of the sample. A long string indicates high ropiness.
Astringency	Similar feeling to very unripe fruit.	If the sample dries out your mouth, it means high astringency.
Mouth coating	The extent to which the product coats the palate and teeth during mastication.	Distribute the product in your mouth and swallow it. If it leaves a coating in your mouth, it is high in mouth coating.
Mouth thickness	Sensation of sample consistency in mouth.	Evaluate the product’s resistance when swallowed with normal speed without tasting the sample.
Smoothness	The smoothness against the palate as it breaks up during mastication.	Perceive the smoothness of the sample by squeezing it between palate and tongue.
Acetic	Acidic smell of vinegar.	Hold your nose to perceive acidic flavor.
Cardboard	Aromatic associated with slightly oxidized fats, reminiscent of wet cardboard packaging.	Tasting of the sample.
Fatty/creamy Foamy	Feeling associated with heavy whipping cream. Foam appearance of the sample.	Compare the product with the given full fat cream (38%) sample. Visual evaluation of the sample.
Powdery/chalky	Powder sensation in mouth.	Visual evaluation of the sample.
Pudding-like	Similar structure to a pudding.	Visual evaluation of the sample.
Shininess	How shiny the surface of the product looks like.	Visual evaluation of the sample.

## Appendix B. Acidification Time in Fermented Soy, Oat, and Coconut

**Table A2:** Fermentation time in hours required to reach pH 4.5 in all fermented bases.

Culture Combination	Base		
	Soy	Oat	Coconut
YF-L01	7.09 ^Ac^	8.57 ^Bd^	5.32 ^C^
YF-L02	6.64 ^Ab^	7.38 ^Bbc^	5.11 ^C^
BY-01	6.91 ^Abc^	7.94 ^Bc^	5.70 ^C^
YF-L01+LGG^®^	6.27 ^Aa^	7.27 ^Bb^	5.33 ^C^
YF-L02+LGG^®^	6.02 ^Aa^	6.65 ^Ba^	5.43 ^C^
BY-01+LGG^®^	6.05 ^Aa^	6.80 ^Bab^	5.93 ^C^

^ABC^ Means with different uppercase superscripts indicate significant differences between different columns (*p* < 0.05). ^abc^Means in the same column with different lowercase superscripts indicate significant differences between different culture combinations (*p* < 0.05)

## Appendix C. Untargeted Volatile Organic Compounds in Each Fermented Base

Untargeted VOCs in fermented soy, oat, and coconut samples in which different culture combinations had an effect.

**Table A3:** Fermented soy samples.

Culture	Ketones	Esters		Terpene		Aldehyde	Furan
	2,3-Pentanedione	Ethyl-Octanoate	Ethyl-Decanoate	Limonene	α-Pinene	Hexanal	2-pentyl-furan
YF-L02	11,281 ^b^	1621 ^b^	973 ^b^	242 ^b^	91 ^c^	203 ^c^	46,421 ^b^
BY-01	4012 ^a^	<LOD	14 ^a^	55 ^a^	17 ^a^	25 ^a^	22,223 ^ab^
YF-L01 + LGG^®^	5439 ^a^	13 ^a^	15 ^a^	113 ^a^	77 ^bc^	33 ^a^	46,281 ^b^
YF-L02 + LGG^®^	11,270 ^b^	14 ^a^	22 ^a^	49 ^a^	50 ^b^	115 ^b^	4887^a^
BY-01 + LGG^®^	14,537 ^b^	1046 ^b^	833 ^b^	79 ^a^	50 ^b^	106 ^b^	25,638 ^ab^

^abcd^ Means in the same column in each fermented base with different lowercase superscripts indicate significant differences between different culture combinations (*p* < 0.05). < LOD means that the results were below the levels of detection of the specific compound. Contents are represented as signal-to-noise values.

**Table A4:** Fermented oat samples.

Culture Combination	Ketones	Ester	Terpene	Aldehyde	Alcohol	
	2,3-Pentanedione	Ethyl Acetate	Limonene	Furfural	1-Hexanol	2-Propanol
YF-L01	54,063 ^d^	1498 ^ab^	18 ^a^	10 ^ab^	59 ^ab^	724 ^b^
YF-L02	12 ^b^	1095 ^a^	41 ^b^	12 ^ab^	120 ^b^	115 ^a^
BY-01	43,895 ^c^	409 ^a^	24 ^a^	19 ^b^	47 ^ab^	6 ^a^
YF-L01 + LGG^®^	430 ^a^	448 ^a^	17 ^a^	13 ^ab^	15 ^a^	123 ^a^
YF-L02 + LGG^®^	154 ^a^	752 ^a^	25 ^a^	9.5 ^ab^	22 ^ab^	8.5 ^a^
BY-01 + LGG^®^	211 ^a^	2205 ^b^	21 ^a^	8.5 ^a^	44 ^ab^	14 ^a^

^abcd^ Means in the same column in each fermented base with different lowercase superscripts indicate significant differences between different culture combinations (*p* < 0.05). <LOD means that the results were below the levels of detection of the specific compound. Contents are represented as signal-to-noise values.

**Table A5:** Fermented coconut samples.

Culture Combination	Ketone		Terpene	Lactone	
	2-Pentanone	2-3-Pentanedione	α-Pinene	γ-Nonalactone	Butyrolactone
YF-L01	11 ^a^	89 ^c^	76 ^bc^	2153 ^ab^	13 ^ab^
YF-L02	8.5 ^a^	66 ^b^	64 ^ab^	1884 ^ab^	12 ^ab^
BY-01	8.0 ^a^	55 ^b^	54 ^a^	1381 ^a^	8.0 ^a^
YF-L01+LGG ^®^	27 ^b^	24 ^a^	70 ^abc^	2212 ^b^	18 ^bc^
YF-L02+LGG ^®^	29 ^bc^	18 ^a^	87 ^c^	2404 ^b^	22 ^c^
BY-01+LGG ^®^	39 ^c^	15 ^a^	66 ^abc^	2153 ^ab^	14 ^ab^

^abcd^ Means in the same column in each fermented base with different lowercase superscripts indicate significant differences between different culture combinations (*p* < 0.05). <LOD means that the results were below the levels of detection of the specific compound. Contents are represented as signal-to-noise values.
